# A blueprint for the implementation of a validated approach for the detection of SARS-Cov2 in clinical samples in academic facilities

**DOI:** 10.12688/wellcomeopenres.15937.2

**Published:** 2020-10-21

**Authors:** Sushmita Sridhar, Sally Forrest, Iain Kean, Jamie Young, Josefin Bartholdson Scott, Mailis Maes, Joana Pereira-Dias, Surendra Parmar, Matthew Routledge, Dominic Sparkes, Lucy Rivett, Gordon Dougan, Michael Weekes, Martin Curran, Ian Goodfellow, Stephen Baker

**Affiliations:** 1 Cambridge Institute of Therapeutic Immunology & Infectious Disease (CITIID), Department of Medicine, University of Cambridge, Cambridge, UK; 2 Wellcome Sanger Institute, Hinxton, UK; 3 Department of Medical Genetics, University of Cambridge, Cambridge, UK; 4 Public Health England Diagnostic Laboratory, Addenbrooke's Hospital, Cambridge, UK; 5 Infectious Diseases, Addenbrooke's Hospital, Cambridge, UK; 6 Division of Virology, Department of Pathology, University of Cambridge, Cambridghe, UK

**Keywords:** COVID-19, SARS-Cov2, diagnostic PCR, qPCR, sample workflow, validation

## Abstract

The COVID-19 pandemic is expanding at an unprecedented rate. As a result, diagnostic services are stretched to their limit, and there is a clear need for the provision of additional diagnostic capacity. Academic laboratories, many of which are closed due to governmental lockdowns, may be in a position to support local screening capacity by adapting their current laboratory practices. Here, we describe the process of developing a SARS-Cov2 diagnostic workflow in a conventional academic Containment Level 2 laboratory. Our outline includes simple SARS-Cov2 deactivation upon contact, the method for a quantitative real-time reverse transcriptase PCR detecting SARS-Cov2, a description of process establishment and validation, and some considerations for establishing a similar workflow elsewhere. This was achieved under challenging circumstances through the collaborative efforts of scientists, clinical staff, and diagnostic staff to mitigate to the ongoing crisis. Within 14 days, we created a validated COVID-19 diagnostics service for healthcare workers in our local hospital. The described methods are not exhaustive, but we hope may offer support to other academic groups aiming to set up something comparable in a short time frame.

## Introduction

SARS-Cov2, the viral agent of COVID-19, is a recent introduction into the human population, and the disease epidemic is expanding nationally (within the UK) and internationally at an unprecedented rate ^1, 2^. As a result, national diagnostic services are stretched and there is a need for alternative laboratory facilities to provide additional diagnostic capacity. The screening of asymptomatic individuals and healthcare workers (HCWs) will be key for controlling the epidemic and also to ensure HCW are a) working in safe conditions with functional personal protective equipment (PPE), b) not transmitting SARS-Cov2 to vulnerable patients on wards, and c) can return to work if they are not actively infected ^3^. However, in almost all cases, embedding a rapid testing workflow for screening asymptomatic individuals and HCWs within the current hospital structure would add additional pressure upon an already overstretched diagnostic service.

The UK government and other organisations have recognised the critical role of additional screening ^4^. For example, the UK has recently initiated the establishment of national testing centres for HCWs; however, there is a need for guidance on how such systems be standardised or scaled. The expected turnaround times from sampling collection to results being reported back to the affected HCW is also a key issue. Whilst central screening facilities will ultimately be beneficial in curtailing the epidemic, smaller academic and non-academic laboratories can (and should) contribute to these efforts. Critically, local facilities (academic laboratories in proximity to or within healthcare facilities) can frequently provide a quicker turnaround time than larger remote facilities due to simpler sampling and shipping logistics or even overstretched, onsite diagnostic laboratories; current turnaround time is typically >48 hours from sample being taken to provision of result. Such delays can have a negative impact on healthcare provision, for example staffing levels may be strained due to HCWs isolating as a result of a respiratory illnesses other than COVID-19. Therefore, there is an urgent unmet need to establish local screening workflows for HCWs and those working in essential service industries.

One of the key limitations associated with the expansion of diagnostics to tackle the COVID-19 outbreak is the availability of protocols, or a scheme, that can be used in suitable laboratories for detecting SARS-Cov2 in relevant clinical samples. In the given circumstances, a robust diagnostic test for COVID-19 should be able to generate a result rapidly, but also maintain a high level of reproducibility, specificity, and sensitivity ^5^. The test also needs to be conducted on an easily accessible clinical sample, such as a dual nose and throat swab, urine, or blood. Or in those with more severe illness sputum or bronchoalveolar lavage. Such tests can be based upon a direct amplification assay for a component of the viral genome, a suitable biomarker or metabolic signature, or the measurement of an indicative acute antibody response. Given a paucity of reliable alternatives, a PCR based approach is currently the most suitable and scalable model, whilst providing an acceptable compromise between turnaround time and accuracy.

The key issues for rapidly establishing a new diagnostic testing platform are sampling, safety, reagents, cleanliness, methodology, and reporting. Early indications for COVID-19 infections is that there are relatively high titres of virus in the respiratory tract, possibly in the gastrointestinal tract, but lower concentrations in blood ^6^. Consequently, nose and throat swabs are widely accepted as the optimal clinical sample for HCWs and others who are likely to have a higher occupational exposure risk and potential upper respiratory tract symptoms to the virus through their work. These swabs need to be handled safely, so the use of a sampling method that inactivates the virus rapidly is essential to protect the sampler and those handling the sample, including couriers, and laboratory staff ^7^. The availability and the expense of reagents required for an effective testing programme at a specific scale (e.g. hospital, company, or local community) is critical given the ongoing demand for specific kits. Tests that require expensive mainstream reagents, or those in short supply, should be avoided where possible. PCR diagnostics are prone to issues with contamination and appropriate workflow and strict sample/reagent segregation needs to be adopted, which may be problematic in some settings. Methodologies and equipment are variable, but every attempt should be made to ensure the tests are performed using a standardised and validated test with appropriate controls. Lastly, the resulting data needs to be authenticated by a qualified individual and reported in a timely fashion through an existing and official reporting system, whilst at the same time ensuring patient confidentiality.

Here we describe our experience in establishing a COVID-19 diagnostics laboratory in an academic containment level 2 (CL2) research facility (UK) in which we validated and established a real-time PCR workflow to detect SARS-CoV2 in nose and throat swabs from HCWs. We developed an assay and workflow over eight working days (set-up to validation to screening) that can produce a quantitative diagnostic result ~4 hours after swabbing.

## Methods

### Swabbing

For the swabbing of known COVID-19 patients and HCWs we developed a kit that can be easily assembled and provided in bulk. Not only does this significantly reduce PPE usage, but also reduces the need for significant interaction between those running the testing clinic and potential CoVID-19 positive HCWs. The kit contained: swabbing instructions, an individually packed sterile swab that can be broken (VWR), a labelled sample tube containing lysis buffer, and gloves (see *Extended data:* Protocol 1).

The instructions indicate the individual to put on the gloves, remove the sterile swab from the packet and swab the back of throat and then the nasal cavity (one swab, two sites). The swab is placed into the labelled sample tube (4ml long necked externally threaded cryovials (Nunc 379146; to avoid aerosols), and the end is submerged in 500µl lysis buffer (4M guanidine thiocyanate (Merck) in 25 mM Tris-HCl, 0.5% β-mercaptoethanol (Sigma), and carrier RNA (100 µl of 1 µg/µl stock; Qiagen). The swab is snapped carefully to avoid disturbing the buffer, and the cap is placed back onto tube containing the buffer and swab and tightened. The tube is gently agitated to ensure even distribution of lysis buffer and labelled with an ethanol resistant pen. The outside of the tube is sprayed with 80% ethanol, placed into a zip lock bag (Onecall) and sealed. One glove is removed, and the zip lock bag is sprayed with 80% ethanol while being held in gloved hand and then passed to a clean hand. The sealed bag is placed in a secure biohazard labelled container for dispatch to a certified CL2 laboratory.

### Nucleic acid extraction

The combination of 4M guanidine thiocyanate and 0.5% β-mercaptoethanol should ensure complete lysis and deactivation of the virus, but to ensure additional safety, the samples are received and unpacked in a class II microbiological safety cabinet (MSC) (see *Extended data:* Protocols 2 and 4) ^8^. Notably, whilst this process should be conducted in a sterile and clean environment with routine cleaning sessions, given the nature of the samples this room is isolated as “a dirty room” and all molecular reagents kept elsewhere. Those working in this room should not enter the room in which molecular reagents are kept and laboratory clothing remains restricted to this room.

The class II MSC should be running as ‘safe’ prior to work to ensure a stabile airflow. The cabinet is cleaned sequentially with 5% bleach, 80% ethanol, and RNaseZap (Sigma). The class II MSC cabinet should be set up with the required reagents and waste vessels before sample bags are placed directly inside and sprayed with 80% ethanol. Barcodes are scanned for tracking and tubes arranged into batches of ≤24 for extraction, dependent on centrifuge rotor capacity. The sample tubes, still containing the swab, are placed into a rack, 500 µl 100% ethanol (final ethanol concentration 50%) added to each tube one-by-one and incubated at ambient temperature for 10 minutes. Top-up lysis buffer containing the internal extraction and amplification control (25 µl of 10 ^-3^ MS2 (~ 6 × 10 ^4^ PFU/ml) per 10 ml of lysis buffer in this case) is next added to each sample (400 μl to make 35% final ethanol concentration) resulting in a total volume of ~1.4 ml per tube. In total, 600 µl of the media is transferred into a spin column (NBS biologicals) over a 2ml RNase-free collection tube (Thermofisher). To avoid contamination, only one column is open at any one time and filter pipette tips are used for each sample. The tubes are loaded into a microcentrifuge rotor inside the class II MSC, and the aerosol-tight lid closed before returning the rotor to the microcentrifuge. The samples are centrifuged for 30 seconds at 15,000 rpm (two spins are required per sample to load the entire volume of lysis buffer).

All pass-through liquid is discarded into designated liquid collection containers (do not mix with disinfectants containing bleach). 500 µl of wash buffer 1 (1M guanidine thiocyanate in 25 mM Tris-HCl, with 10% ethanol) is added onto the columns and tubes are centrifuged for 30 seconds at 15,000 rpm. The pass-through liquid is discarded, and 500 µl of wash buffer 2 (25 mM Tris-HCl buffer with 70% ethanol) is added and again tubes are centrifuged for 30 seconds at 15,000 rpm. Finally, a second 500 µl of wash buffer 2 is added, and the tube is centrifuged for 2 minutes at 15,000 rpm. The silica spin column is transferred to a new collection tube and centrifuged at 15,000 rpm for 1 minute to remove residual ethanol. The silica spin column is transferred to a new RNase free tube with the appropriate sample label. 100 µl of nuclease free water is added to each column and left to stand for 1 minute before centrifugation for 1 minute at 15,000 rpm. The spin columns are discarded, and the eluted samples are either directly taken for qRT-PCR or an aliquot is frozen at -20°C for subsequent amplification. The remaining nucleic acid extracts are frozen at -80°C with the location recorded on the ‘sample record’ form.

### Amplification of SARS-Cov2 nucleic acid

Once the nucleic acid (viral RNA) has been extracted, it can be amplified to detect SARS-Cov2 (see *Extended data:* Protocol 3). Notably, this work should be done in a “clean room,” and the operators should wear laboratory clothing that is restricted to this room. Movement to other working areas where biological or molecular contamination may be an issue should be restricted, and there should be no access to the dirty room.

Per reaction, the master mix is made up of: 12.5 µl 2X Luna Universal Probe One-Step reaction mix, 0.5 µl of 20 pmoles/µl Wu forward primer (ATGGGTTGGGATTATCC **T**AAATGTGA), 0.5 µl of 20 pmoles/μl Wu reverse primer (GCAGTTGT **G**GCATC **T**CC **T**GATGA **G**), 0.3 µl of 10pmoles/µl MGB Probe 3 FAM (ATGCTTAG **A**AT **T**ATGGCCTC **A**C), 0.5 µl of 10 pmoles/μl of internal control forward primer (MS2) (supplied by Eurogentec), 0.5 µl of 10 pmoles/µl internal control reverse primer (MS2), 0.3 µl of 10 pmoles/µl internal probe (MS2 ROX), 1 µl of Luna WarmStart RT Enzyme Mix (New England Biolabs) and 3.9 µl water. Once the master mix is prepared, it can be stored at 4°C short term or -20°C longer term. If using immediately, 20 µl can be aliquoted into each well of a 96-well plate in a clean Class II cabinet and then combined with 5 µl of each RNA extract, using a different pipette tip for each well. Ideally, the master mix preparation and addition of RNA to each well should be done in separate class II cabinets to minimize contamination.

The MS2 internal extraction and amplification control that underwent the full extraction protocol is included as the negative extraction control in a minimum of two wells on the qRT-PCR plate. To check for contamination in the qRT-PCR process, 5 µl nuclease-free water (minimum 2 wells) is included as the qRT-PCR negative control. 5 µl of spiked SARS-Cov2 template plasmid is included in a single well as the qRT-PCR positive control. After adding 5 µl of each sample to its designated well, the plate is sealed carefully with an optically clear plastic seal. The plate is centrifuged for 1 minute at 1,000 rpm at 4°C and then inserted in the qRT-PCR machine (QuantStudio, Thermofisher scientific) and the run is parametrised. FAM and ROX are acquired; ROX is used to detect the internal control; FAM is used to detect SARS. The assay is run for 2 minutes at 25°C, 15 minutes at 50°C (for the reverse-transcriptase), 2 minutes at 90°C, before 45 cycles of 95°C for 3 seconds followed by 60°C for 30 seconds.

The results are determined by confirmation of the correct positive controls (amplification of the spiked target), the extraction and amplification controls of all samples (ROX channel), no amplification in the negative controls, and consistent mean values of controls. SARS-Cov2 positivity is confirmed by amplification in the FAM channel with an appropriate (non-undulating or linear) sigmoidal curve with a CT value ≤36. The CT values of MS2 and MGB probe 3 are maintained in a Levey-Jennings plot to track quality and reproducibility of the assay ^9, 10^.

## Establishing and validating the workflow in our setting

### Establishing a workflow for SARS-Cov2 qRT-PCR

Upon the decision to rapidly establish the qRT-PCR assay we identified several challenges, and these included: a) establishment and validation of a method suitable for diagnostic reporting, b) safe extraction of nucleic acid from a highly transmissible virus, c) accessing reagents required for performing extractions and amplifications, d) establishing a “clean” diagnostic workflow to minimise the risk of contamination, and e) creating a system in which HCWs could be swabbed and the data reported confidentially within a specified timeframe.

### Setting up a diagnostic qRT-PCR

**Figure 1.**
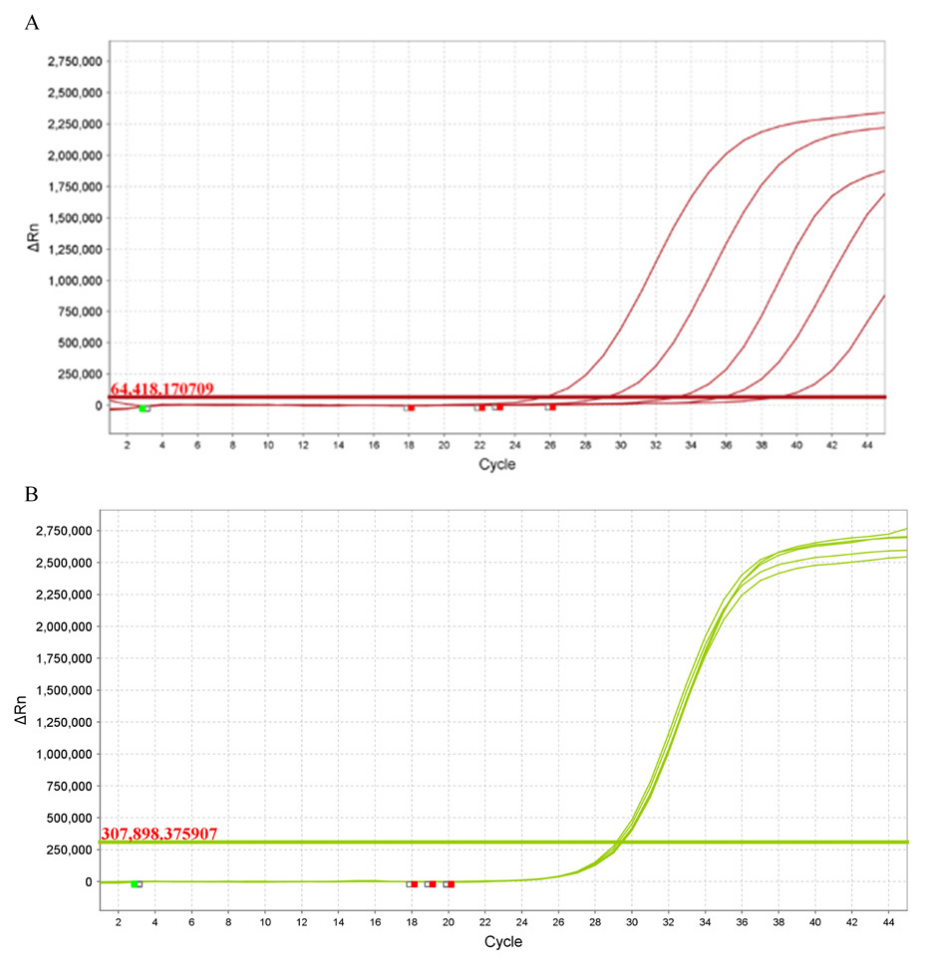
Establishing positive and negative qRT-PCR control for SARS-Cov2. ( **A**) Amplification plot of cloned SARS-Cov2 template plasmid in 5 10-fold dilutions with FAM reporter. The x-axis displays the number of PCR cycles and the y-axis show the magnitude of normalized fluorescence signal generated by the reporter at each cycle during the PCR amplification in the form of ∆Rn. ( **B**) Amplification plot of cloned MS2 control from spiked test samples with ROX reporter. The x-axis displays the number of PCR cycles and the y-axis show the magnitude of normalized fluorescence signal generated by the reporter at each cycle during the PCR amplification in the form of ∆Rn. Data analysed using QuantStudio 6 and 7 Flex Realtime PCR System Software colours correspond to plate location.

In our setting, diagnosis of infections for the hospital is normally performed in the region Public Health England (PHE) diagnostic laboratory, which is co-manned by hospital and PHE staff, serving our and local hospitals. Upon agreement with senior diagnostic staff we sought their approval to duplicate their in-house generated, validated assay on our equipment. The diagnostic laboratory provided access to their in-house method (designed by Martin Curran and Surendra Parmar) and provided a collection of anonymised SARS-Cov2 positive extractions (as determined by the same PCR method) and a cloned positive control. The required reagents were ordered and the QuantStudio machine calibrated to run the qRT-PCR. qRT-PCR was initially performed using existing positive samples and ten-fold dilutions of the cloned target gene (a conserved region with the ORF1 polyprotein). Upon amplification, we were able to replicate the positive signals from known positive samples (with comparable CT values of between 20 and 33) and generate a reproducible standard curve that could be used for all following amplifications and validations ( Figure 1a). Additionally, during this process we validated the amplification process by the addition of a positive control; MS2 nucleic acid was added to all samples with the exception of the positive SARS-Cov2 control and the negative controls ( Figure 1b). Notably, these assays were run a minimum of three occasions over differing days to assess the degree of experimental variation. Through this procedure of testing, troubleshooting, and assay development, we were able to show reproducible amplifications and have an assay ready for downstream validation.

### Swabbing and nucleic acid extraction

It should be noted that at the time of starting the scheme, samples with the potential to harbour SARS-Cov2 virus were classified by the UK Health and Safety Executive (HSE) as requiring a Containment Level 3 (CL3) laboratory. This level of security was required due to the infectious nature of the virus and potential for airborne transmission. Existing sampling procedures exploited a viral transport media containing ingredients to preserve the virus and restrict the growth of non-viral pathogens. The extraction of nucleic acid (or viral inactivation prior to downstream processes) in a CL3 facility was deemed a major bottleneck that could be circumvented. Consequently, we considered it essential to inactivate the sample safely at source so as to minimise risk.

A protocol was established that was risk assessed by the University Health and Safety Committee to inactivate combined nose and throat swabs immediately after they are taken from the individual being sampled. The protocol is outlined in Methods (for the full protocol see *Extended data:* Protocol 2) and was established from existing methods known to chemically inactivate viruses. We utilised existing data regarding coronavirus and other highly infectious viral pathogens. Several methods, including heat inactivation were considered, but the selected method using a mixture of guanidine thiocyanate and β-mercaptoethanol was considered to be the most suitable for validation. Whilst existing data demonstrated that the designated approach was safe for viral extraction it had not been tested on COVID-19 patients. A recent publication has highlighted that traditional AVL lysis buffer, on which our home-made equivalent is based, does not lead to 100% inactivation of live virus when mixed at a ratio of 4:1 and left with a contact time of 10 minutes. However, our protocol relies on the use of dry swabs, therefore any dilution effect of the lysis buffer is negated ^9^. In addition, because of the locality of the testing laboratory, the minimum contact time between the swab and the lysis buffer is typically >1hr. This was followed by the addition of ethanol to a final concentration of 50% in an MSC.

### Sample workflow

**Figure 2.**
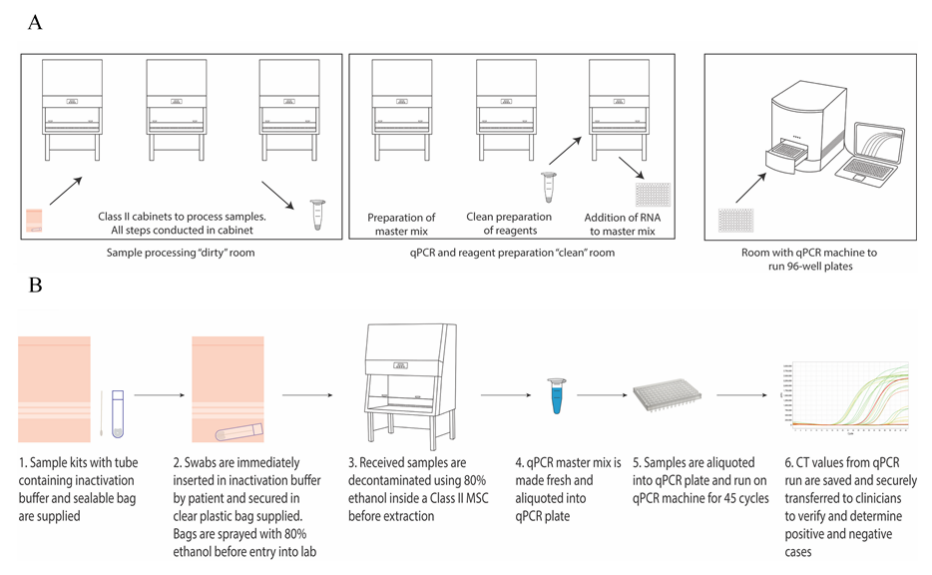
Establishing a diagnostic workflow for qRT-PCR for SARS-Cov2. ( **A**) Diagram displaying the segregation of the “dirty”, “clean” and “amplification” rooms. Note the use of separate cabinets for the preparation of reagents in the “clean” room. Individuals working in the “dirty” or “amplification” rooms are unable to enter the “clean” room on the same working day. ( **B**) Diagram showing a suitable workflow of samples from swabbing to amplification to reporting.

A critical step in establishing a diagnostics facility is the segregation of workspace and staff, preventing the cross contamination of samples, equipment, and reagents. The mode of operation is not typical for many research laboratories where communal facilities are used according to the requirements of the specific project. The research laboratory was reorganised to create “dirty”, “clean”, and amplification areas ( Figure 2a). These were strategically located in separate rooms and a strict regime was created where equipment, staff, PPE, and samples were restricted to these specific rooms. All laboratory staff were trained in the new containment structure and in the assays being performed. This component involved the transfer of materials, knowledge, and protocols between PHE Cambridge and the research laboratory. Laboratory staff were given specific roles and were restricted to the “clean” or “dirty” work areas for a single working day. Ultimately, we had developed a workflow that could be tested for screening sample from COVID-19 patients ( Figure 2b).

### Final validation of qRT-PCR assay from known COVID-19 patients

**Figure 3.**
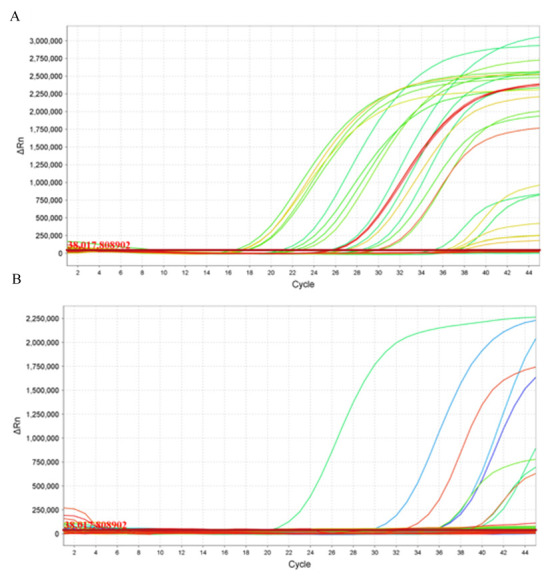
Validating and introducing a qRT-PCR for SARS-Cov2. ( **A**) Amplification plot of SARS-Cov2 from clinical samples from known COVID-19 patients. Data generated following the entire process on blinded swabs. The x-axis displays the number of PCR cycles and the y-axis show the magnitude of normalized fluorescence signal generated by the reporter at each cycle during the PCR amplification in the form of ΔRn. ( **B**) Amplification plot of SARS-Cov2 from samples taken from healthcare workers on first day of screening. The x-axis displays the number of PCR cycles and the y-axis show the magnitude of normalized fluorescence signal generated by the reporter at each cycle during the PCR amplification in the form of ΔRn. Data analysed using QuantStudio 6 and 7 Flex Realtime PCR System Software, colours correspond to plate location.

The next stage in validating the process was to run the full extraction protocol and assays on samples from patients that had previously tested positive for SARS-Cov2 in the assay performed by the hospital diagnostic laboratory. Buffers and extraction kits were constructed in the “clean” rooms and provided for patient sampling. In agreement with the hospital, for the purposes of developing a diagnostic test, we approved that a group of 20 known COVID-19 patients and a group of 20 individuals assumed not to be infected with SARS-Cov2 would be screened. Consequently, 40 swabs were taken from these individuals according to the protocol; these were dispatched to the laboratory for processing and analysis. The samples were anonymised, and research workers were blinded from knowing which samples were positive or negative. Additionally, instead of a precise 20/20 split in the provided samples, 19 were from known SARS-Cov2 patients and 21 from uninfected patients. Again, this was not revealed to the staff performing the assay until after the tests results were known. Data from this experiment are shown in Figure 3a and Table 1. There was 100% correlation between the test results initially generated by the diagnostic laboratory and the research laboratory, with 19 generating CT values of between 16 and 36, and 21 generating no detectable signal. All controls were as expected. At this point the assay was repeated several times to be further validated for reproducibility. Specifically, the initial 40 extracted RNA samples provided by the hospital were assessed by qRT-PCR on our system three times to determine reproducibility of the qRT-PCR signal. In addition, further testing RNA samples were provided by the hospital as known positives and negatives for use. A total of ~ 30 of these samples were analysed by qRT-PCR to check for robustness and concordance of our qRT-PCR assay. Upon completion of these validation samples, the assay was offered to the hospital for the screening of HCWs.

**Table 1.** Assessment of known patient samples.

Sample	Hospital assessment	Lab protocol assessment	Lab protocol CT
**1**	**Positive**	**Positive**	**17**
**2**	**Positive**	**Positive**	**18**
**3**	**Positive**	**Positive**	**18**
**4**	**Positive**	**Positive**	**18**
**5**	**Positive**	**Positive**	**18**
**6**	**Positive**	**Positive**	**21**
**7**	**Positive**	**Positive**	**22**
**8**	**Positive**	**Positive**	**22**
**9**	**Positive**	**Positive**	**23**
**10**	**Positive**	**Positive**	**26**
**11**	**Positive**	**Positive**	**26**
**12**	**Positive**	**Positive**	**27**
**13**	**Positive**	**Positive**	**28**
**14**	**Positive**	**Positive**	**29**
**15**	**Positive**	**Positive**	**29**
**16**	**Positive**	**Positive**	**30**
**17**	**Positive**	**Positive**	**30**
**18**	**Positive**	**Positive**	**35**
**19**	**Positive**	**Positive**	**36**
**20**	**Negative**	**Negative**	**> 36**
**21**	**Negative**	**Negative**	**> 36**
**22**	**Negative**	**Negative**	**> 36**
**23**	**Negative**	**Negative**	**> 36**
**24**	**Negative**	**Negative**	**> 36**
**25**	**Negative**	**Negative**	**> 36**
**26**	**Negative**	**Negative**	**> 36**
**27**	**Negative**	**Negative**	**> 36**
**28**	**Negative**	**Negative**	**> 36**
**28**	**Negative**	**Negative**	**> 36**
**30**	**Negative**	**Negative**	**> 36**
**31**	**Negative**	**Negative**	**> 36**
**32**	**Negative**	**Negative**	**> 36**
**33**	**Negative**	**Negative**	**> 36**
**34**	**Negative**	**Negative**	**> 36**
**35**	**Negative**	**Negative**	**> 36**
**36**	**Negative**	**Negative**	**> 36**
**37**	**Negative**	**Negative**	**> 36**
**38**	**Negative**	**Negative**	**> 36**
**39**	**Negative**	**Negative**	**> 36**
**40**	**Negative**	**Negative**	**> 36**

### Provision of testing for healthcare workers

Within two weeks of the start of the process, the screening procedures were approved by the hospital and made available for hospital staff through occupational health. A firewall was built between the hospital and the research laboratory to protect confidential data without losing track of samples. A system was created where samples and data could be managed within a single system through use of a unique identifier number and barcode, hence there was a logged transfer of the samples to the research laboratory, where samples could be tested, and data reported within the research laboratory. The hospital established a swabbing pod and offered structured screening to staff, and a depository for samples was established at a single point within the hospital. Samples were transported securely to the research laboratory in a risk assessed, spill proof container by selected courier, which was a member of clinical staff involved in the project. Data from the first screening run is shown in Figure 3b and permitted the detection of several positives. The CT values from these ranged from 18–36 and the turnaround time was ~4 hours from sample arrival to result being available for reporting. However, given the scheduled sampling, we aimed to report the data within 24 hours. Using our 96-well plate setup and allowing for multiple negative and positive controls per plate, we were able to assay and report up to 90 patient samples per qRT-PCR run.

Data were checked and validated prior to reporting by a senior member of laboratory staff. All CT values and curves were checked, and the presence of amplification in the controls was verified. All data were entered into the official hospital database and verified by a clinical virologist prior to reporting back to occupational health. Residual RNA samples were suitable for downstream analysis and we were able to contribute to ongoing COVID-19 genome sequencing projects affiliated to COG-UK.

### Troubleshooting

Clearly establishing an assay rapidly in difficult circumstances requires frequent validation, reappraisal, and troubleshooting. As the project developed, more levels of management, oversight, and communication were brought in. For example, within the hospital, links had to be established between those working within the wards and occupational health to determine HCW populations for priority and routine screening. Thus ethical, logistical, and practical barriers had to be identified and managed.

Within the laboratory setting, potential for the contamination of materials was a key consideration that had to be managed. For example, at one stage, background levels of amplification on negative samples were elevated above acceptable levels, which was assessed to be contamination. Based on the controls used in the specific plate (having negative and positive extraction controls, swabs extracted using two different kit batches, qRT-PCR negative and positive controls), we hypothesized that the contamination was occurring in the QuantStudio equipment. This was potentially due to SARS-Cov2 DNA that was being amplified inside the machine and causing all samples to have CT values ~32. Consistency in CT value suggested that the issue was at the amplification stage and not at the extraction or qRT-PCR preparation stage.

The QuantStudio comes with a Background Calibration plate as part of its calibration system. This plate was checked to assess whether the background profile had changed substantially. If the machine “passed” the calibration, we assessed whether the profile of the background fluorescence differed from when it was previously calibrated. We performed a background calibration plate run and then the bottom plate of the machine was cleaned sequentially with 10% bleach, 95% ethanol, and MilliQ water. The baseplate and optical plate were removed from the machine for deep cleaning. The baseplate was rinsed in 10% bleach, followed by MilliQ water, 95% ethanol, then water again. Liquid was aspirated from the wells, which were then wiped with a lens cleaner tissue. The upper optical plate was wiped with cotton swabs containing 95% ethanol in case any dust particles were occluding the surface.

A further background calibration plate was run after cleaning and several wells were found to have high readings. To revalidate the machine, a plate of 20 µl mastermix plus water (negative controls) in any “problem” wells (to rule out the possibility of well-specific amplification/contamination) and additional wells scattered around the plate were assessed. Based on the location of the problem wells, contamination was often more severe at the edges of the plate, so these were also checked. The rest of the wells of the 96-well plate were filled with 10 µl of water only. The location of the “problem” wells suggested that there might have been a failed plate seal at some point, which may have released some DNA into the machine to amplify. This plate was found to give low background; several positive samples and positive and negative controls were run, and the contamination issues were found to be resolved.

## Discussion

In the continuing public health crisis, we need as much capacity as possible for supporting diagnostic services to ensure key workers and HCW are screened frequently. This places an enormous pressure on an already saturated system. The introduction of large screening services will play a huge role in tackling the epidemic in the UK and elsewhere but lacks some of the speed and flexibility that small on-site diagnostic laboratories can provide. We recognised the need to repurpose our laboratory for COVID-19 screening; this was initiated without request to provide some additional local capacity. Many academic facilities may be in a similar position but may be unsure about how to start proceedings and what regulations are in place. We suggest that groups establish the assay and processes so they can be prepared as the need arises. The route we describe here is not a scalable solution to the international lack of diagnostic testing, but a blueprint for what can be established in a standard academic research laboratory within 14 days. At the time of writing we have a full sample workflow from swabbing to diagnostic testing of HCWs at our healthcare facility, with the capacity of approximately 100 tests a day with a result provided within 24 hours. This number of tests can be expanded, but is dependent on maintaining enough extraction rooms, key staff, and of course key reagents. We sought to develop a test that works independently of kits from major suppliers, but there will potentially be issues with other resources as the crisis develops. The theoretical turnaround time is 4 hours, but this is dependent on integration with occupational health facilities and the diagnostic laboratory and ensuring there is a sustainable communication and enough staff to maintain the process.

In setting up this process there are many challenges and pitfalls, especially given the time constraints of providing a functional service that can be rapidly deployed, and we recognise that everything described here is not exhaustive. Many laboratories differ in equipment, facilities, capacity, expertise and staffing; additionally, being in close proximity to a major infectious disease centre with an excellent diagnostic facility is a major advantage. However, the methods and stages of laboratory repurposing described will, we hope, be of value to other academic laboratories in the UK and internationally that are aiming to make a useful contribution. Particularly, with some simple modifications and training we feel that this could be developed and rolled out in low and middle-income countries, providing vital molecular capacity for this and future epidemics.

In summary, the key problems to solve are safety, reagents, cleanliness, methodology, validation, and reporting. Here, we tackled new challenges on an almost daily basis, but inactivating the virus on contact improved the process and ensured the swabs could be extracted safely in a CL2 laboratory. Access to reagents is key, and we suggest that groups become less reliant on kits from major manufacturers, unless essential. This step reduces costs and puts less pressure on existing supply chains of key kits and equipment ^11^. Cleanliness is paramount, and sample flow, room segregation, and dedicated staff are essential. Having a reliable diagnostic facility that you can partner with will reduce many of the initial issues. These groups, such as the PHE laboratory here, provided excellent advice, reagents, methodology, and support for set up. Having access to good clinical facilities for validating the assay is essential; the whole process (from swab to report) needs to be comprehensively tested before being rolled out. Lastly, reporting needs to be conducted with the provision of experienced staff, again a link with clinical diagnostic facility is essential.

Here we provide a brief outline of our experience in establishing a COVID-19 diagnostic laboratory in a standard molecular bacteriology laboratory, which we hope is useful to other groups in a similar position. It was achieved under challenging circumstances through the collaborative efforts of scientists, clinical, and diagnostic staff with the ability to generate something constructive that we hope will contribute to the ongoing crisis.

## Data availability

### Underlying data

All data underlying the results are available as part of the article and no additional source data are required.

### Extended data

Open Science Framework: A blueprint for the implementation of a validated approach for the detection of SARS-Cov2 in clinical samples in academic facilities: extended data, https://doi.org/10.17605/OSF.IO/VZ7UD ^12^.

This project contains the following extended data:

- Protocol 1. CoVID-19 Swabbing procedure
- Protocol 2. Extraction procedure
- Protocol 3. qRT-PCR plate setup protocol
- Protocol 4. Buffer Preparation
- Risk assessment

Data are available under the terms of the Creative Commons Zero "No rights reserved" data waiver (CC0 1.0 Public domain dedication).
