# Supplementary material for: A blueprint for the implementation of a validated approach for the detection of SARS-Cov2 in clinical samples in academic facilities
Source: Wellcome Open Res. 2020 Oct 21;5:110. Originally published 2020 Jun 1. [Version 2] doi: 10.12688/wellcomeopenres.15937.2 (PMC7590889; doi:10.12688/wellcomeopenres.15937.2)
Supplement: Peer review history

# Peer review: Reviewer response for version 2

**DOI:** 10.21956/wellcomeopenres.17996.r41025

**Published:** 2020-10-22

**Authors:** Kondwani C. Jambo

**Affiliations:** 1 Malawi-Liverpool-Wellcome Trust Clinical Research Programme, University of Malawi College of Medicine, Blantyre, Malawi

The authors have adequately addressed my queries. I have no further comments.

 I fully approve the manuscript.

Is the rationale for developing the new method (or application) clearly explained?

Yes

Is the description of the method technically sound?

Yes

Are the conclusions about the method and its performance adequately supported by the findings presented in the article?

Yes

If any results are presented, are all the source data underlying the results available to ensure full reproducibility?

Partly

Are sufficient details provided to allow replication of the method development and its use by others?

Yes

Reviewer Expertise:

NA

I confirm that I have read this submission and believe that I have an appropriate level of expertise to confirm that it is of an acceptable scientific standard.

# Peer review: Reviewer response for version 2

**DOI:** 10.21956/wellcomeopenres.17996.r41026

**Published:** 2020-10-21

**Authors:** Brendan W. Wren

**Affiliations:** 1 Department of Infection Biology, London School of Hygiene and Tropical Medicine, London, UK

The authors have made appropriate changes and the paper should be indexed as soon as possible.

Is the rationale for developing the new method (or application) clearly explained?

Yes

Is the description of the method technically sound?

Yes

Are the conclusions about the method and its performance adequately supported by the findings presented in the article?

Yes

If any results are presented, are all the source data underlying the results available to ensure full reproducibility?

Yes

Are sufficient details provided to allow replication of the method development and its use by others?

Yes

Reviewer Expertise:

Micobial pathogenesis and application to diagnostics and vaccine development.

I confirm that I have read this submission and believe that I have an appropriate level of expertise to confirm that it is of an acceptable scientific standard.

# Peer review: Reviewer response for version 1

**DOI:** 10.21956/wellcomeopenres.17481.r40503

**Published:** 2020-09-21

**Authors:** Brendan W. Wren

**Affiliations:** 1 Department of Infection Biology, London School of Hygiene and Tropical Medicine, London, UK

The article is a true and necessary blueprint for the rapid set up of facilities for the diagnosis of Covid-19 infection. The article is most timely and provides all the necessary details that many university/research centre laboratories with PCR thermal cycling facilities could adopt. This should prove very useful, for example enabling universities to offer regular testing of students and staff, relieving the pressure on national health care systems.

 The protocols (including sampling, safety, reagents, cleanliness, methodology and reporting) are very well written, carried out to the highest of standards and are of sufficient detail to be widely adopted.

 A useful section was on contamination with background levels of nucleic acid amplification on negative samples. This was traced to the QuantStudio equipment which was subsequently stripped and cleaned. An alternative proof of the source of contamination would have been to use a different machine.

 The conclusions fully supported the findings presented. A statement on the capacity and throughput of the set up would have been useful, presumable this is dependent on the 96-well plate format.

 Given the current pandemic and capacity difficulties encountered by many countries in testing for the virus, it is imperative that this article should be indexed as soon as possible.

Is the rationale for developing the new method (or application) clearly explained?

Yes

Is the description of the method technically sound?

Yes

Are the conclusions about the method and its performance adequately supported by the findings presented in the article?

Yes

If any results are presented, are all the source data underlying the results available to ensure full reproducibility?

Yes

Are sufficient details provided to allow replication of the method development and its use by others?

Yes

Reviewer Expertise:

Micobial pathogenesis and application to diagnostics and vaccine development.

I confirm that I have read this submission and believe that I have an appropriate level of expertise to confirm that it is of an acceptable scientific standard.

# Peer review: Reviewer response for version 1

**DOI:** 10.21956/wellcomeopenres.17481.r40368

**Published:** 2020-09-15

**Authors:** Kondwani C. Jambo

**Affiliations:** 1 Malawi-Liverpool-Wellcome Trust Clinical Research Programme, University of Malawi College of Medicine, Blantyre, Malawi

Sridhar *et al. *describe their experience in setting up and validating a COVID-19 diagnostic service in an academic setting. They provide extremely useful lessons and SOPs from sample collection, processing, diagnosis, to reporting. 

 However, I have some queries and suggestions for the authors. 

Queries:

1. Do the authors have any direct data on the efficiency of their virus inactivation protocol?
2. What are the key recommendations that would help minimise the QuantStudio contamination?
3. Can the authors comment on the potential for incorporating sample pooling into their workflow? 

Suggestions:

1. The authors should consider presenting the validation data in Figure 3 in the form of a table, in addition to the amplification plots.
2. The authors should provide the data to support this statement "At this point, the assay was repeated several times to be further validated for reproducibility before being offered to the hospital for the screening of HCWs". This would help QA and QC for those who want to adopt the setup. 

Is the rationale for developing the new method (or application) clearly explained?

Yes

Is the description of the method technically sound?

Yes

Are the conclusions about the method and its performance adequately supported by the findings presented in the article?

Yes

If any results are presented, are all the source data underlying the results available to ensure full reproducibility?

Partly

Are sufficient details provided to allow replication of the method development and its use by others?

Yes

Reviewer Expertise:

Immunology, biomedical laboratory science

I confirm that I have read this submission and believe that I have an appropriate level of expertise to confirm that it is of an acceptable scientific standard, however I have significant reservations, as outlined above.
